# Macrophage Migration Inhibitory Factor Promotes the Interaction between the Tumor, Macrophages, and T Cells to Regulate the Progression of Chemically Induced Colitis-Associated Colorectal Cancer

**DOI:** 10.1155/2019/2056085

**Published:** 2019-07-10

**Authors:** Thalia Pacheco-Fernández, Imelda Juárez-Avelar, Oscar Illescas, Luis I. Terrazas, Rogelio Hernández-Pando, Carlos Pérez-Plasencia, Emma B. Gutiérrez-Cirlos, Federico Ávila-Moreno, Yolanda I. Chirino, José Luis Reyes, Vilma Maldonado, Miriam Rodriguez-Sosa

**Affiliations:** ^1^Biomedicine Unit, Facultad de Estudios Superiores Iztacala, Universidad Nacional Autónoma de México (UNAM), Tlalnepantla C.P. 54090, Mexico; ^2^Experimental Pathology Section, National Institute of Medical Sciences and Nutrition “Salvador Zubirán”, Tlalpan, C.P. 14000 Mexico city, Mexico; ^3^Epigenetics, National Institute of Genomic Medicine, Tlalpan, C.P 14610 Mexico city, Mexico

## Abstract

Colitis-associated colorectal cancer (CRC) development has been shown to be related to chronically enhanced inflammation. Macrophage migration inhibitory factor (MIF) is an inflammatory mediator that favors inflammatory cytokine production and has chemotactic properties for the recruitment of macrophages (Møs) and T cells. Here, we investigated the role of MIF in the inflammatory response and recruitment of immune cells in a murine model of chemical carcinogenesis to establish the impact of MIF on CRC genesis and malignancy. We used BALB/c MIF-knockout (MIF^−/−^) and wild-type (WT) mice to develop CRC by administering intraperitoneal (i.p.) azoxymethane and dextran sodium sulfate in drinking water. Greater tumor burdens were observed in MIF^−/−^ mice than in WT mice. Tumors from MIF^−/−^ mice were histologically identified to be more aggressive than tumors from WT mice. The localization of MIF suggests that it is also involved in cell differentiation. The relative gene expression of *il-17*, measured by real-time PCR, was higher in MIF^−/−^ CRC mice, compared to the WT CRC and healthy MIF^−/−^ mice. Importantly, compared to the WT intestinal epithelium, lower percentages of tumor-associated Møs were found in the MIF^−/−^ intestinal epithelium. These results suggest that MIF plays a role in controlling the initial development of CRC by attracting Møs to the tumor, which is a condition that favors the initial antitumor responses.

## 1. Introduction

Cancer is one of the major causes of death worldwide; in 2015, it caused 8.8 million deaths, and the number of new cases is expected to increase by approximately 70% in the next two decades. Colorectal cancer (CRC) is the third most frequent neoplasm in the world, and the third leading cause of global mortality among human cancers (774,000 deaths) [1].

Several epidemiological studies have demonstrated a direct relationship between chronic inflammatory diseases and the increased risk of developing cancer [2], for example, gastritis caused by *Helicobacter pylori* and gastric cancer [3, 4], Barrett's esophagus and esophageal cancer [5], and chronic pancreatitis and pancreatic cancer [6, 7]. Additionally, 18% of patients with inflammatory bowel disease (IBD), which includes ulcerative colitis and Crohn's disease, develop CRC [5].

Chemokines and proinflammatory molecules are determining factors in CRC biology that attract and activate immune cells that exert antitumor responses, and there is an increasing interest in developing therapies against the disease [8]. In this context, MIF is a cytokine that plays an important role as a regulator of innate and adaptive immunity [9] with a significant influence on the activation of the inflammatory cascade [10] but also has chemotactic properties to recruit Møs and T cells [11, 12]. Hence, MIF has been proposed as a possible therapeutic target for colorectal cancer [13].

MIF is secreted by a variety of immune cells, such as T and B cells, monocytes, macrophages (Møs), dendritic cells (DCs), and granulocytes [14], and nonimmune cells, such as epithelial cells in direct contact with the environment [15]. MIF is released in response to antigenic challenges, such as lipopolysaccharide (LPS) or gram-positive exotoxins, to cytokines such as TNF-*α* and IFN-*γ*, as well as physiological stress [16]. Moreover, MIF is an activator of monocytes, Møs, and DCs [17–19] and increases phagocytosis and the destruction of intracellular pathogens [20]. The importance of MIF for inflammatory conditions is reflected by the high levels of this cytokine found in patients with diabetes mellitus [19, 21], rheumatoid arthritis [22], multiple sclerosis [23], atherosclerosis [24], asthma [25], inflammatory liver disease [26], ulcerative colitis [27], and cancer [28, 29].

MIF is constitutively expressed in epithelial cells from the intestinal tract [15], and its expression is enhanced during ulcerative colitis, promoting inflammation and the development of severe pathology [27]. MIF-knockout (MIF^−/−^) or wild-type (WT) mice treated with anti-MIF therapy did not develop ulcerative colitis, or ulcerative colitis was significantly reduced in these mice [30, 31], while transgenic mice that overexpressed MIF developed more severe colitis [32].

Although chronic ulcerative colitis may precede CRC development [33, 34], there is not enough evidence supporting the hypothesis that the high levels of MIF present in chronic colitis are the trigger for the subsequent development of CRC. A previous study, where the colon 26 cancer cell line was inoculated in the mice, showed that the treatment with anti-MIF antibody reduced the tumor size and the angiogenesis in it [13], whilst the transplantation of the CT-26 colon carcinoma cell lines and subsequent administration of anti-MIF antibodies or the synthetic MIF inhibitor ISO-1 resulted in the reduction of tumor volume [29]. *In vitro* studies demonstrate that the use of small interfering RNA (siRNA) to knock down the MIF mRNA reduced the migration of the colon 26 cells [13], and the addition of recombinant MIF increased the invasiveness and expression of related genes in LoVo cells [29]. These observations are supported by a mouse model of small intestinal tumorigenesis in MIF^−/−^ mice, where the MIF-deficient mice developed less adenomas compared to MIF-sufficient mice [35]. However, these observations differ from those in patients with advanced stages of CRC, whose higher concentrations of MIF correlate with a survival greater than 5 years, which is significantly higher than the survival of patients with lower MIF concentrations [34].

It is well known that various solid tumors overexpress MIF and notably high levels of MIF are found in serum, epithelial cells, and liver metastases in CRC patients and murine models [29, 36, 37]. Although previous studies were carried out in mice lacking the MIF gene (MIF^−/−^) or using synthetic MIF inhibitors, cell lines implanted in these mice produced high levels of MIF, meaning that the initial immune response was influenced by MIF. Thus, it is necessary to elucidate the role of MIF in the CRC genesis and malignancy in a model completely free of MIF.

Here, we use a murine model of chemically induced colitis-associated colorectal cancer wherein tumors develop from the mouse's cells (not by implantation of transformed cells), to determine the influence of MIF in the beginning, as well as in the development of the tumors. Furthermore, we analyzed the influence of MIF in the modeling of the tumor microenvironment.

## 2. Materials and Methods

### 2.1. Animals

Six- to eight-week-old female BALB/c mice were purchased from Harlan Laboratories (ENVIGO, Mexico) and maintained in a pathogen-free environment at the Facultad de Estudios Superiores- (FES-) Iztacala, Universidad Nacional Autónoma de Mexico (UNAM), Mexico. MIF^−/−^ mice were developed as described previously and backcrossed for >10 generations to a BALB/c genetic background [38]. Animals were housed and maintained in a pathogen-free environment at our animal facility in accordance with institutional and Mexican Regulations of Animal Care and Maintenance (NOM-062-Z00-1999, 2002) and the US National Institutes of Health Guidelines for the Care and Use of Laboratory Animals.

### 2.2. Chemical Colitis-Associated Colorectal Cancer Development

**Figure 1:**
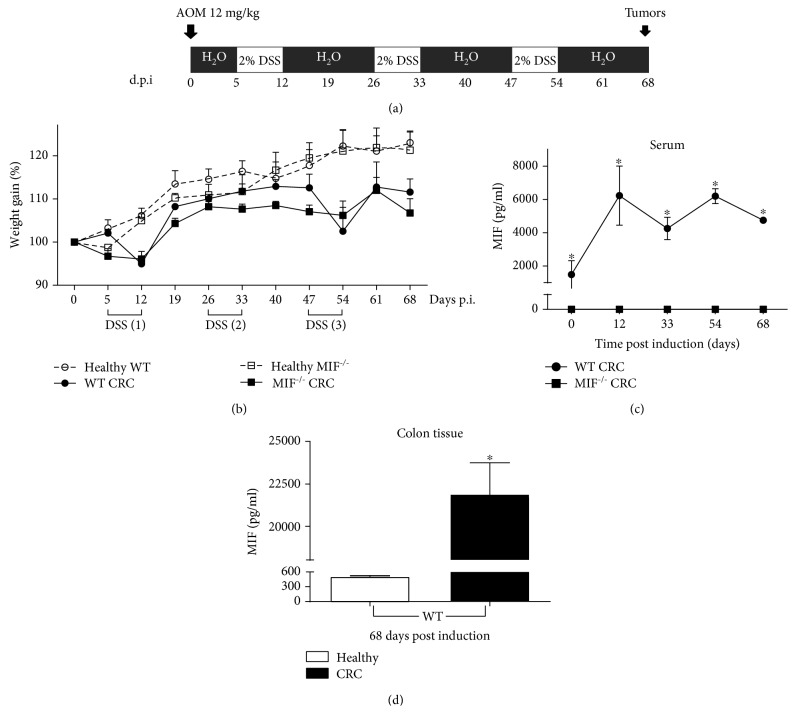
Chemical colitis-associated cancer increases serum MIF levels. (a) Both WT and MIF*^−/−^* female BALB/c mice were injected via intraperitoneal injection with AOM; then, 3 cycles of 2% DSS were administered in drinking water, each followed by 14 days of water free of DSS. (b) Weight gain was supervised during cancer development. MIF concentrations (c) in blood serum over time (after each DSS cycle at 12, 33, 54, and 68 days) and (d) in colonic tissue after 68 days were measured by ELISA. Data are representative of three independent experiments and are plotted as the means (±SEM), *n* = 3 mice per group; ^∗^ *p* < 0.05, ^∗∗^ *p* < 0.01, and ^∗∗∗^ *p* < 0.001. MIF: macrophage migration inhibitory factor; CRC: colorectal cancer; AOM: azoxymethane; DSS: dextran sodium sulfate; d.p.i: days post induction.

A variation of Neufert's chronic-inflammation-derived colorectal cancer model was developed [39]. Briefly, mice were intraperitoneally (i.p.) injected with 12 mg/kg of azoxymethane (AOM, Sigma-Aldrich, St. Louis, MO, USA). Five days later, dextran sodium sulfate (DSS, Alfa Aesar, Ward Hill, MA, USA) dissolved at 2% in drinking water was administered *ad libitum* for 7 days. Afterward, mice were maintained for 14 days with regular water and underwent two more DSS cycles (Figure 1(a)). Four groups were formed: healthy WT, WT mice treated with AOM and DSS (WT CRC), healthy MIF^−/−^ mice, and MIF^−/−^ mice treated with AOM and DSS (MIF^−/−^ CRC).

Mice were euthanized 68 days after AOM injection under CO_2_/O_2_ excess atmosphere, and all efforts were made to minimize suffering. The colon was excised, and the length from the distal caecum to the anus was measured. Fecal matter was flushed out with cold PBS, 100 U of penicillin/streptomycin, and 2 mM glutamine (all from GIBCO-BRL, Grand Island, NY, USA), and the colon was opened longitudinally. Tumors were measured with a digital caliper, and size was determined by the following formula: tumor size (mm^3^) = (length × width^2^)/2. Then, the tumor burden per mouse was defined by the addition of all tumor sizes.

### 2.3. MIF Quantification

The MIF concentration was determined in mouse serum and colon tissue. Blood samples were obtained prior to AOM injection and after every DSS cycle from the tail vein, and the serum was obtained by centrifugation at 600 × g in a Prism R microcentrifuge (Labnet International, Woodbridge, NJ, USA). For colon protein extraction, 0.5 cm of healthy or tumor tissue was excised after mouse euthanasia at day 68. The tissue was homogenized in RIPA buffer with phosphatase and protease inhibitors (Roche Diagnostics GmbH, Mannheim, Germany) and stored at -70°C until use. Once thawed, the samples were shaken for 30 min at 4°C and centrifuged at 6000 × g for 5 min at 4°C in a Prism R centrifuge (Labnet International). Proteins were precipitated with ice-cold acetone overnight, centrifuged at 9400 × g for 15 min, and solubilized in PBS with protease and phosphatase inhibitors. Fifty microliters of serum or 20 ng of tissue protein was used for MIF quantification using the Mouse MIF DuoSet Sandwich ELISA kit (R&D Systems, Minneapolis, MN, USA) in accordance with the manufacturer's instructions. Samples were read in an Epoch microplate spectrophotometer at 405 nm (BioTek, Winooski, VT, USA).

### 2.4. Histological and Immunohistochemical Analysis

Distal colon longitudinal sections (0.5 cm) were fixed by immersion in 4% buffered-paraformaldehyde, dehydrated with increasing concentrations of ethanol, embedded in paraffin, and cut into 4 *μ*m sections. The sections were stained with hematoxylin and eosin (H&E) to determine cell infiltration, the grade of dysplasia, polyp type, and crypt morphology. Sections were also stained with Alcian blue and contrasted with H&E to make goblet cells apparent, allowing us to determine goblet cell loss and the tumor cell differentiation level. To determine the localization of macrophages and MIF within the tumor, specific purified antibodies were used (anti-F4/80 (BioLegend, London, UK) and anti-MIF (Santa Cruz Lab, CA, USA)). Slides were stained with the Histostain Bulk Kit (Invitrogen, Carlsbad, CA, USA) according to the manufacturer's instructions. Macrophages in the tumor stroma and epithelial tissue were counted. All specimens were evaluated by a blinded histopathologist using light microscopy (UNICO, Princeton, NJ, USA).

### 2.5. RNA Isolation and RT-PCR

Tumor and healthy colon sections (0.5 cm) were homogenized in TRIzol reagent (Thermo Fisher Scientific, Waltham, MA, USA), and RNA extraction was performed according to the manufacturer's instructions. cDNA was synthesized with the Maxima First Strand cDNA Synthesis for RT-qPCR kit (Thermo Fisher Scientific), and RT-PCR was performed using KAPA Taq (Kapa Biosystems, Woburn, MA, USA). The relative expressions of *mif*, *il-17*, *il-18*, *tnf-α*, *il-10*, *il-4*, *il-1β*, *foxp3*, *arginase-1*, and *inos* transcripts were determined and compared to the housekeeping gene *β-actin* (Table 1).

### 2.6. Lamina Propria Cell Isolation and Flow Cytometry Analysis

Colon sections were carefully flushed with cold PBS + 1x penicillin-streptomycin-glutamine (Gibco) and opened longitudinally. The tissue was then incubated in HBSS containing 2 mM EDTA, 2% FBS, and 1x penicillin-streptomycin-glutamine cocktail (Gibco) for 30 min at 37°C in a water bath shaker at 250 rpm. Afterward, the tissue was washed, minced with razor blades, and digested in a solution of 2 mg/ml collagenase type IV (Sigma-Aldrich), 40 *μ*g/ml DNase I, and 10% FCS in DMEM for 60 min at 37°C in a shaker at 250 rpm. Lamina propria cells were isolated using 100 *μ*m and 40 *μ*m Falcon cell strainers (Becton Dickinson, Sunnyvale, CA). Mononuclear cells were further isolated with a Percoll (MP Biomedicals, Irvine, CA, USA) gradient separation method. Briefly, cells were resuspended in 40% Percoll-DMEM, underlayered with 70% Percoll-DMEM and centrifuged at 1000 × g for 20 min. The interface was collected for FACS analysis. Lamina propria mononuclear cells were stained with fluorescently labeled anti-CD3, anti-CD4, and anti-CD8 antibodies for T cells, anti-F4/80 for macrophages, anti-CD11b for myeloid cells, anti-Gr1 for MDSCs, anti-Ly6G for granulocytes, and Zombie Aqua for cell viability. All antibodies were used according to the manufacturer's instructions (BioLegend). An Attune NxT (Thermo Fisher Scientific) flow cytometer was used to quantify cell populations, and FlowJo v10 was used for FACS analysis.

### 2.7. Statistical Analysis

The data were analyzed either by a one-way ANOVA followed by Tukey's multiple comparison test or by an unpaired Mann-Whitney test with GraphPad Prism 6 (San Diego, CA) software, with a significance of *p* < 0.05.

## 3. Results

### 3.1. Chemically Induced Colitis-Associated Cancer Increases Serum MIF Levels

MIF has been shown to favor inflammatory responses and cancer-favoring mechanisms in previous experimental murine models of CRC, but none of these models was completely devoid of MIF. This study was conducted to determine colitis-associated CRC development in the absence of systemic MIF.

During CRC development, physical symptoms were monitored. MIF^−/−^ CRC mice exhibited piloerection since the first DSS cycle and bloody diarrhea since the second DSS cycle, whereas the WT CRC mice showed moderate symptoms after the second DSS cycle (data not shown). The body weight was measured weekly over the course of the AOM/DSS treatment. Both the MIF^−/−^ CRC and WT CRC groups showed a decrease in weight after the first and third cycles of DSS compared to their respective healthy controls. Mice from CRC groups gained weight slowly, but they did not recover the weight of the mice in healthy groups after 40 days (Figure 1(b)).

In order to validate the lack of MIF in knockout mice in our model, we evaluated the systemic concentration of MIF in WT and MIF^−/−^ mice before and over time post CRC induction. As shown in Figure 1(c), serum MIF levels from healthy WT mice (time 0) (1487 ± 8 pg/ml) consistently increased after the first (6235.89 ± 1781.15 pg/ml) and third (6204.66 ± 737.82 pg/ml) cycles of DSS. After the second cycle of DSS and on sacrifice day, the MIF peripheral concentrations (4258.9 ± 1253.646 pg/ml and 4748.606 pg/ml, respectively) remained increased compared to those of healthy mice but to a lesser extent than the other measurement points. We demonstrated the absolute lack of MIF in the MIF^−/−^ mice; serum concentrations were undetectable throughout CRC development.

Typically, in humans as in mice, under at rest healthy conditions, the basal levels of MIF in serum or plasma are higher than for other cytokines [40]. However, under stressful conditions, their levels increase rapidly, promoting acute and chronic inflammation, and are related to cancer. To confirm MIF production by WT tumors, we evaluated the protein concentration in situ by ELISA. At day 68 post CRC induction, the level of MIF protein in colonic tissue of WT CRC (21905 ± 9047 pg/ml) was increased approximately 42 times compared to that of the baseline MIF level in healthy WT mice (513 ± 15 pg/ml) (Figure 1(d)). MIF protein was not detected in MIF^−/−^ mice without or with CRC (data not shown).

### 3.2. MIF Deficiency Facilitates Increased Tumor Development

To determine the colorectal cancer severity, the colon was obtained and cut longitudinally to expose the intestinal lumen and the length was measured at 68 days post CRC induction.

**Figure 2:**
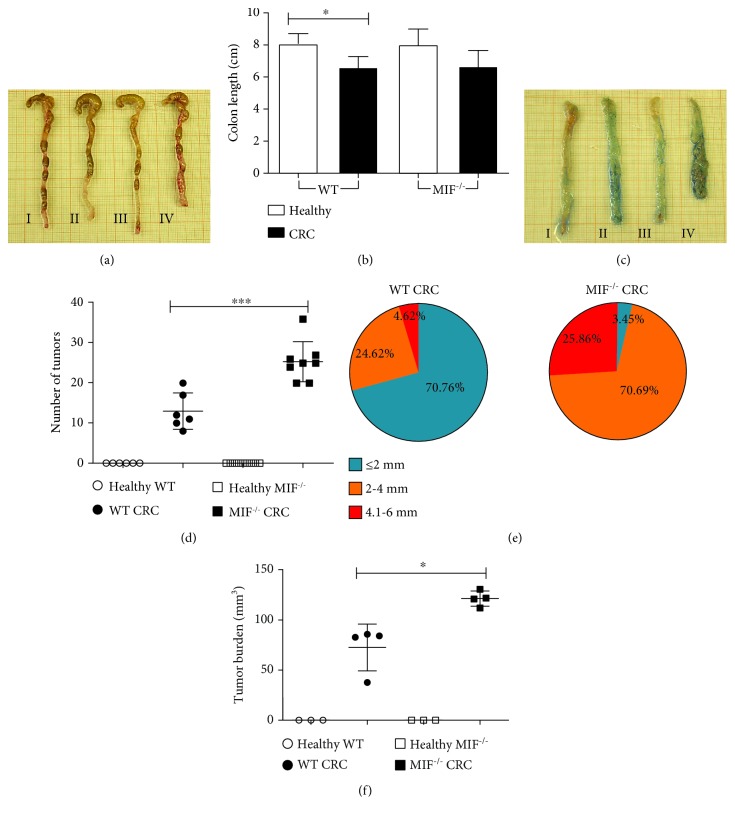
MIF deficiency facilitates increased tumor development. After 68 days post induction (d.p.i). (a) Colons from (I) healthy WT, (II) WT CRC, (III) healthy MIF^−/−^, and (IV) MIF^−/−^ CRC mice were obtained and (b) measured from caecum to anus. Then, (c) the colons were opened longitudinally to measure (d) tumor number and (e) diameter to determine (f) tumor burden. Data are representative of three independent experiments and are plotted as the means (±SEM), *n* = 3 mice per group; ^∗^ *p* < 0.05, ^∗∗^ *p* < 0.01, and ^∗∗∗^ *p* < 0.001. MIF: macrophage migration inhibitory factor; CRC: colorectal cancer; AOM: azoxymethane; DSS: dextran sodium sulfate; d.p.i: days post induction.

The length of the healthy colon was similar in healthy WT (8.09 ± 0.74 cm) and healthy MIF^−/−^ (8.04 ± 1.06 cm) mice. WT CRC mice presented significantly shorter colons than WT healthy mice (Figure 2(a)); the colon length was 6.64 ± 0.46 cm and 7.17 ± 0.68 cm long, respectively (Figure 2(b)).

The number and size of the tumors are indicative of cell transformation and proliferation. The AOM/DSS-induced CRC model in the BALB/c mice causes the development of multiple tumors in the medial and distal zones of the colon, but not in the proximal area (Figure 2(c)). MIF^−/−^ mice developed 24 ± 3 tumors per colon, twice the number of tumors developed by WT mice (12 ± 5 tumors per colon) (Figure 2(d)). Additionally, these tumors were larger: 70.76% of the WT tumors were below 0.5 mm in diameter and only 4.62% exceeded 4 mm; in contrast, most of the tumors (70.69%) in the MIF^−/−^ colon were between 2 and 4 mm in diameter and 25.86% of the tumors exceeded 4 mm (Figure 2(e)). The increased tumor development in MIF-deficient mice was also reflected in the tumor burden. MIF^−/−^ CRC mice developed 121.396 ± 1.03 mm^3^ of tumoral tissue per colon versus 72.63 ± 12.22 mm^3^ in the WT CRC mice (Figure 2(f)).

### 3.3. MIF-Deficient Mice Have a Worse Prognosis than WT Mice

**Figure 3:**
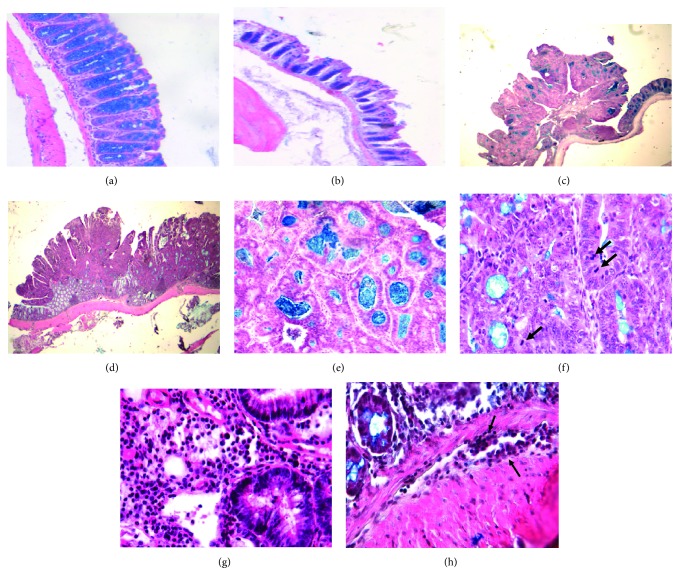
MIF-deficient mice have worse tissue damage than WT mice. Representative comparative micrographs of MIF^−/−^ and WT lesions. (a) Normal histological structure of colonic mucosa from WT mice. (b) In comparison to those in WT mice, colonic crypts are shorter in MIF^−/−^ mice. (c) Polypoid well-differentiated adenocarcinoma in WT mice. (d) MIF^−/−^ mice show larger tumors than WT mice. (e) Tumors from WT mice are composed of neoplastic cells organized in well-differentiated glands, many of which show detached necrotic cells and abundant Alcian blue-positive material that correspond to mucin, denoting well-differentiated adenocarcinoma. (f) In contrast, MIF^−/−^ mice showed neoplastic glands with more stratified epithelium, reduced mucin production, and numerous mitotic figures (arrows) denoting a lower differentiation grade. (g) High-power micrograph of the base from the tumor that developed in WT mice, showing well-differentiated glands revisited by neoplastic epithelial cells exhibiting a large hyperchromatic nucleus. The surrounding stroma shows numerous inflammatory cells and distended blood vessels. (h) In contrast, the base of the tumor that developed in MIF^−/−^ mice shows smaller mildly differentiated glands, the surrounding tissue (smooth muscle) shows scarce inflammatory infiltration and neoplastic cuboidal cells, and some of these cells are in the lumen of blood vessels denoting hematogenous invasion (arrows). Photographs are representative of three independent experiments, *n* = 3 mice per group, and samples were analyzed by a blinded histopathologist.

Tumor morphology determines the malignancy and prognosis of cancer. By staining tumor samples with hematoxylin/eosin and Alcian blue, we compared colon sections from healthy and CRC groups (Figure 3). Compared to healthy WT (Figure 3(a)), healthy MIF^−/−^ colonic mucosa present shorter crypts (Figure 3(b)). In both the WT CRC (Figure 3(c)) and MIF^−/−^ CRC (Figure 3(d)) groups, shorter crypts formed by smaller cuboid epithelial cells were observed and were shortest in the latter group. Larger tumors were observed in the MIF^−/−^ colon (Figure 3(d)). Both CRC groups (Figures 3(e)–3(f)) showed well-differentiated polypoid adenocarcinomas, made up of well-differentiated glands revisited by stratified cylindrical large epithelial cells with atypical hyperchromatic nuclei, mitotic figures, and some detached necrotic cells in the gland lumen. Nonneoplastic epithelium near the polypoid tumors showed hyperplasia and regenerative activity with abundant inflammatory cells in the lamina propria. MIF^−/−^ CRC showed reduced mucin production compared to WT CRC. Compared to WT CRC mice (Figure 3(e)), MIF^−/−^ CRC mice (Figures 3(f) and 3(h)) showed larger tumors with less-differentiated glands containing more stratified epithelium that showed larger nuclei and more numerous mitotic figures that were frequently atypical; in the base of these tumors (Figure 3(g)), there were nodules of polygonal undifferentiated cells that were invading blood and lymphatic vessels, denoting higher malignancy levels in contrast with the tumors observed in WT CRC (Figure 3(h)), which did not exhibit these histological abnormalities. Inflammatory cell infiltration was higher in WT CRC mice than in MIF^−/−^ mice, with more numerous neutrophils and macrophages than the tumors developed in MIF^−/−^ mice (Figures 3(e) and 3(f)).

### 3.4. MIF Is Increased in Epithelial Cells, Especially in Well-Differentiated Cells

**Figure 4:**
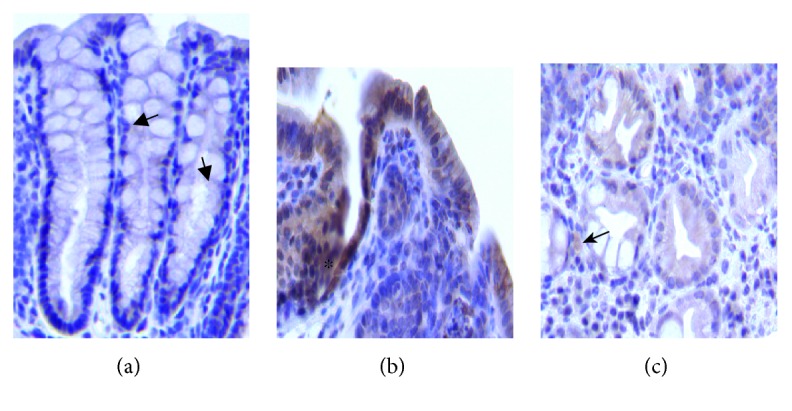
Representative micrographs of MIF detection by immunohistochemistry in WT mice. (a) Colonic epithelium with slight MIF immunostaining (arrows) from the control mice. (b) WT CRC mice showed hyperplastic and regenerative epithelium (∗) with strong MIF immunostaining. (c) Neoplastic well-differentiated glands showed mild MIF immunostaining, while inflammatory cells such as macrophages (arrow) showed stronger immunoreactivity (400x all micrographs). Photographs are representative of three independent experiments, *n* = 3 mice per group, and samples were analyzed by a blinded histopathologist.

The distribution of MIF may reflect its role in tumor development. Thus, sections from the colon were used to detect MIF by immunohistochemistry. WT control mice showed slight MIF immunostaining in the colonic epithelium (Figure 4(a)), while WT CRC mice showed strong MIF immunostaining in inflammatory cells located in the lamina propria, and the staining was stronger in the cytoplasm of regenerating epithelium near the neoplastic polyps (Figure 4(b)). In contrast, neoplastic cells from well-differentiated polypoid adenocarcinoma showed mild MIF immunostaining, while inflammatory cells, particularly macrophages, showed stronger immunoreactivity (Figure 4(c)). Thus, the strongest MIF immunostaining was shown by regenerating hyperplastic epithelium, as well as the associated inflammatory cells, and a progressive decrease of MIF staining was observed in neoplastic cells with stable or increased MIF immunostaining in inflammatory cells.

### 3.5. The Macrophage Population in the Tumor Stroma Is Diminished in the Absence of MIF

**Figure 5:**
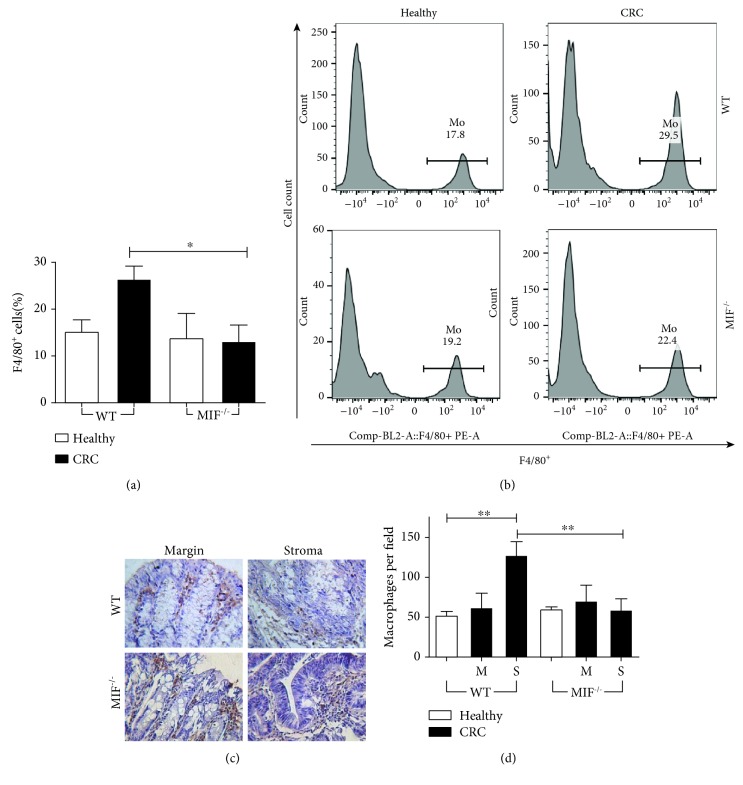
The macrophage population in the tumor stroma is diminished in the absence of MIF. (a) F4/80^+^ CD11b+ macrophage percentage and (b) representative histograms of macrophage staining in the lamina propria from the colon of a mouse with colorectal cancer. (c) Immunohistochemical analysis of macrophages (F4/80^+^ cells) and (d) quantification of the number of macrophages in the tumor margin (M) and tumor stroma (S). Data are representative of three independent experiments and are plotted as the means (±SEM), *n* = 3 mice per group; ^∗^ *p* < 0.05, ^∗∗^ *p* < 0.01, and ^∗∗∗^ *p* < 0.001.

MIF can bind to CXCR2 in macrophages and may induce the recruitment of these cells. MIF is also an important immune modulator, and inflammation-derived tumors are greatly influenced by the immune system. Therefore, we examined the percentage of macrophages within the tumor from the parent gate (big and complex cells) and the macrophages per field in both the tumor margin and the tumor stroma (Figure 5). Flow cytometric analysis (Figure 5(a)) showed that the percentages of F4/80^+^ cells in healthy WT and healthy MIF^−/−^ mice were similar (13.35 ± 4.45% and 13.97 ± 5.23%, respectively), demonstrating that our MIF^−/−^ mouse model is viable and comparable to other models, with no deficiencies in the initial population of macrophages. When WT mice developed CRC, the percentage of macrophages increased dramatically (26.62 ± 6.87%), but the macrophage increase was not observed in the MIF^−/−^ CRC mice (13.27 ± 9.13), which showed percentages similar to those of the healthy controls. Representative histograms of every group are shown in Figure 5(b).

The findings above were supported by immunohistochemical analyses. F4/80^+^ cells were stained, and the number of events per field in the tumor margin and in the tumor stroma was counted (Figure 5(c)). An increase in macrophages was only observed in the tumor stroma of WT CRC mice (128 ± 48 counts per field) but not in the tumor margin (62.5 ± 17.52 counts per field). This effect was not observed in the MIF^−/−^ CRC mice; the number of macrophages in the tumor margin (70.6 ± 19.39 counts per field) was similar to the number in the tumor stroma (59.43 ± 13.52). F4/80^+^ events in MIF^−/−^ CRC mice were comparable to those in both WT and knockout healthy mice (52.60 ± 4.686 and 60.50 ± 2.398 counts per field, respectively) (Figure 5(d)).

The data reported above show that a higher number of macrophages were found in the WT mouse tumor stroma than in the MIF-deficient mouse tumor stroma after AOM/DSS treatment.

### 3.6. T Cell Percentage Is Not Affected by the Absence of MIF

MIF is a chemotactic molecule for T cells because of its affinity for the CXCR4 receptor.

**Figure 6:**
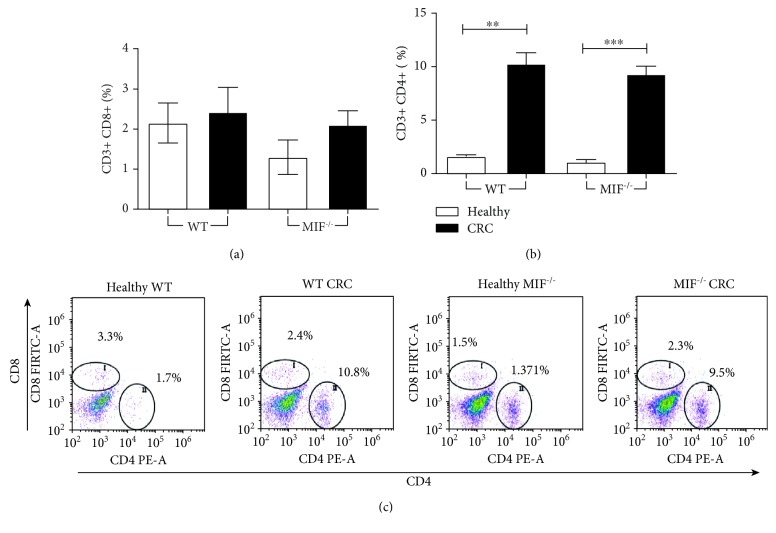
The T cell percentage is not affected by the absence of MIF absence. (a) CD8+ vs. (b) CD4+ T cell percentage and (c) representative dot plots of T cell staining in the lamina propria from the colon of a mouse with colorectal cancer. Data are representative of three independent experiments and are plotted as the means (±SEM), *n* = 3 mice per group; ^∗^ *p* < 0.05, ^∗∗^ *p* < 0.01, and ^∗∗∗^ *p* < 0.001.

We analyzed the percentage of CD4+ and CD8+ T cell populations by flow cytometric analysis (Figure 6). CD4+ and CD8+ cells were selected from CD3+ live cells taken from the lymphocyte population. Nonsignificant differences were found in CD8+ T cell populations among all the groups at 68 d.p.i. (Figure 6(a)). In contrast, AOM/DSS treatment caused an increase in CD4+ T cells at 68 days after AOM injection. WT CRC (10.31 ± 2.66%) and MIF^−/−^ CRC (9.34 ± 1.91%) mice showed higher percentages of CD4+ T cells than their healthy controls (WT 1.621 ± 0.1413% vs. MIF^−/−^1.097 ± 0.2190%). However, there were no differences due to the MIF^−/−^ genotype (Figure 6(b)). In Figure 6(c), representative dot plots taken from the T cell analysis with FlowJo are shown.

### 3.7. MIF Deficiency Does Not Change the Th17 Cytokine Profile or the Activation of Macrophages in the Colon

**Figure 7:**
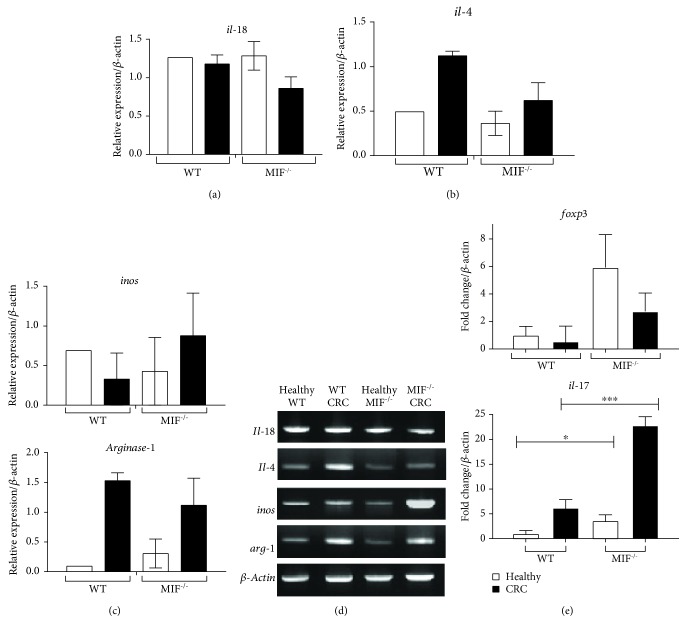
MIF deficiency promotes Th17 cytokine profile development and the alternative activation of macrophages in the colon. Transcripts of molecules related to the tumor microenvironment profile were analyzed by RT-PCR. In this figure, we show the expressions of (a) *il-18*, (b) *il-4*, and (c) *inos* and *arginase-1* relative to the housekeeping gene *β-actin*. (d) Representative electrophoresis gels are shown to show differences between groups. (e) Real-time PCR measurement of relative gene expressions of *foxp3* and *il-17*. Data are representative of two independent experiments and are plotted as the means (±SEM), *n* = 3 mice per group; ^∗^ *p* < 0.05, ^∗∗^ *p* < 0.01, and ^∗∗∗^ *p* < 0.001.

Because MIF is an inflammation promoter that activates the transcription of inflammatory cytokines in macrophages and because of the role of macrophages in T cell activation and tumor microenvironment modulation, we analyzed the relative expression of genes related to the function of immune cells and macrophage polarization (Figure 7).

We observed the relative expressions of *il-18* (Figure 7(a)), *il-4* (Figure 7(b)), and *inos* and *arginase-1* (Figure 7(c)) compared to *β-actin* expression by endpoint RT-PCR. Representative electrophoresis gels are observed in Figure 7(d). No statistical differences were found in the expressions of these genes. Also, *foxp3* and *il-17* (Figure 7(e)) were analyzed by real-time PCR; we observed an increase in the transcription of the *il-17* gene in MIF^−/−^ CRC samples (22.86 ± 1.744‐fold change) compared to WT CRC samples (6.226 ± 1.634‐fold change). No differences were found in *tnf-α*, *il-10*, and *tfg-β* (Supplementary Figure 1).

**Figure 8:**
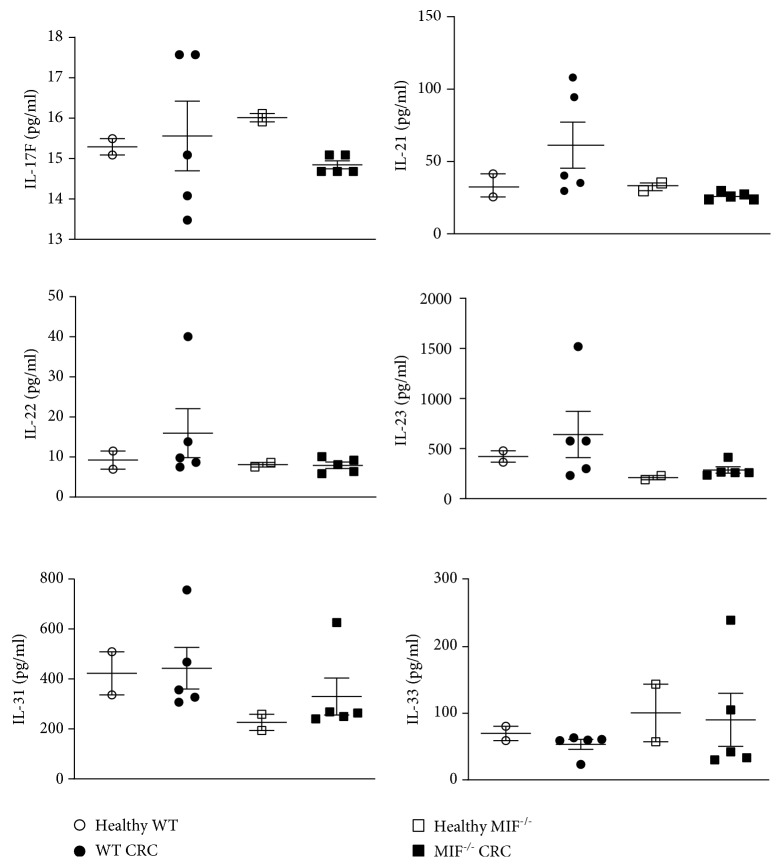
Expressions of Th17-related cytokines. The expressions of IL-17F, IL-21, IL-22, IL-23, IL-31, and IL-33 were detected by ELISA from colonic protein. Healthy and CRC proteins from WT and MIF^−/−^ mice were analyzed. Data are representative of one experiment and are plotted as the means (±SEM), *n* = 2 mice per control group, and *n* = 5 mice per treated group.

Other Th17-related molecules were detected by ELISA (Figure 8). We did not find statistical significance among groups in the concentration of IL-17F, IL-21, IL-22, IL-23, IL-31, and IL-33.

## 4. Discussion

The role of MIF as a possible target therapy for CRC has been suggested by different previous studies [13]; however, MIF possibly plays a favorable role in CRC for some patients [41]. The present study shows a different perspective on the role of MIF in modeling the tumor microenvironment. By analyzing macrophages and T cell populations infiltrating murine colorectal tumors, we demonstrated the participation of MIF in tumor cell differentiation and the fight of the immune system against tumor development.

First, we demonstrated the complete lack of MIF in our knockout model and its increased presence during WT tumor development. All the MIF^−/−^ mice showed undetectable levels of MIF both in serum and in situ, despite CRC induction. From here on, we show the role of MIF in a completely MIF-free system. MIF concentrations in a WT CRC colon were found to be increased approximately 42 times compared to those in a healthy WT colon. A similar proportion was reported by He et al. in CRC patients: these authors found 20-40 times more MIF-positive cells in colon carcinoma tissue than in normal tissue [29]. The increase of MIF in tumors shows the attempt of the immune system to defend the host against abnormal cell development, but this role has not been reported previously because there were no murine models that showed it.

MIF distribution can help us identify the role of MIF in tumor development. Consistent with previous reports [15], in our model, MIF expression was widely distributed in lymphoid tissue and even muscle tissue, but the majority of the expression was found in epithelial cells. In WT CRC tumors, MIF expression was most evident in hyperplastic and regenerative colonic epithelium and early neoplastic lesions and decreased as neoplastic cells organized to form glands. Neoplastic cell differentiation is related to the malignancy level [42, 43] and correlated with our histological observations of less-differentiated neoplastic glands denoting higher malignancy in MIF^−/−^ mice, which exhibited reduced MIF immunostaining. These observations correlate with those in esophageal squamous cell carcinoma where VEGF, IL-8, and MIF can be correlated with tumor cell differentiation [44] and with cell differentiation in early embryos [45]. MIF expression can be upregulated in response to growth factors and promote cell proliferation of colon cancer cells (colon-26) [46] and, according to our results, in the active regenerative colonic epithelium. The suggested mechanism for this upregulation is the interaction between JAB1 protein and MIF, where MIF inactivates JNK activity, hence blocking AP-1 transcriptional activity and maintaining cell cycle arrest [47]. Although MIF absence can explain the less-differentiated and more malignant tumors, it cannot be correlated to the increased proliferation of tumor cells in MIF^−/−^ mice.

Previous studies have demonstrated that MIF induces cellular proliferation by activating the ERK1-ERK2-MAPK [48] and AKT [49] pathways and suppresses JAB1 activity [47] and p53-mediated growth arrest and apoptosis [14, 50]. Other *in vitro* and murine colorectal cancer models have shown that MIF promotes tumorigenesis [35, 51], angiogenesis [51], migration [29, 36, 52–54], and mesenchymal-epithelial transition [55]. Recently, MIF has been proposed as a possible therapeutic target for colorectal cancer [13]. Although this study used MIF^−/−^ mice or synthetic and biological inhibitors of MIF, the CRC model was never devoid of endogenous MIF. CRC cell lines transplanted in mice are major MIF producers, or in the case of MIF inhibition, the genesis of the tumor prior to inhibition is supported by MIF. This is the first paper, to our knowledge, that describes a murine model completely free of MIF protein since the onset of the disease and throughout the development of colorectal cancer.

We evaluated tumor development in the complete absence of MIF. MIF-deficient mice developed twice as many tumors as WT mice; thus, MIF is involved in the control of colorectal cancer development. We counted and measured the tumors after 68 days of CRC induction with azoxymethane; during this time, either the presence of MIF slowed down colorectal tumor development in WT mice or tumor growth was enabled in knockout mice. In the MIF^−/−^ mice, not only the number but also the size of tumors was increased. Even though MIF has been shown to have tumor-promoting properties, the absence of MIF from the onset of the tumor in our model promoted the development of larger and more aggressive tumors. Moreover, the differential histological characterization showed a different growth path and rate dependent on MIF, denoting its influence in modeling the tumor microenvironment. The amount of mucin-producing goblet cells was similar in both healthy groups of mice, showing no initial deficiency due to the lack of MIF in knockout mice. The loss of mucins in the mucosa is related to the development of colorectal-adenocarcinoma rather than tumors being caused by microsatellite instability [56]. Mucin loss is associated with inflammation-derived colon cancer and supports the assumption of the role of the immune system in the tumor microenvironment in our model.

We found that, despite MIF promoting protumorigenic processes, it may also have a beneficial role at some point during colorectal cancer development. Our study may be consistent with the results previously reported in metastatic colon cancer patients; patients with high levels of MIF in their connective tissue had a better survival prognosis than patients with lower MIF concentrations [41].

To determine the mechanism by which MIF may control colorectal cancer development, we analyzed the T cell and macrophage populations. CRC cells have been reported to secrete MIF at concentrations sufficient to attract T lymphocytes to the tumor [57], and MIF can drive macrophage, neutrophil, and T cell migration in a chemokine-like manner [11, 16]. We found significantly fewer macrophages in the tumors of MIF-deficient mice than in those of WT mice by flow cytometry and immunohistochemical analysis; these differences were found in the tumor stroma but not in the tumor margin. The number of macrophages in the tumor margin of MIF-deficient mice was similar to that of healthy mice, showing that the resident macrophages are not affected by the lack of MIF but migration is decreased in the knockout mice. This is in agreement with a previous study, where healthy MIF^−/−^ mice show no difference in the basal number of F4/80^+^ and CD3+ cells compared to WT mice [58]. We demonstrated that MIF participates in the recruitment of macrophages to the tumor site in the murine CRC model. The lack of these cells may be the reason why MIF deficiency enables tumor growth. In patient samples, a high density of CD68+ macrophages in the tumor stromal area was found to be correlated with diminished metastasis to lymph nodes, as well as reduced tumor budding in the invasive margin and increased tumor-free survival [59]. All these data support the idea that MIF influences the initial response against tumor cells, modulating early immune responses. In this line, it has been shown that MIF is necessary for antigen sampling and transport from the gut to the lymph nodes [60], which may decrease the capacity of our MIF^−/−^ mice to control tumor development. Also, MIF-deficient mice cannot properly control microbiota content, related to increased intestinal permeability [58]; this could possibly augment the damage of the mucosa faster than in the WT mice, but this hypothesis is yet to be described.

The tumor stroma is reported to be essential in the progression of colorectal cancer [61] and in determining the polarization of the immune cells therein and thus in modeling the tumor microenvironment [62]. Therefore, we intended to characterize the expression profile of certain genes that encode molecules involved in T cell and macrophage polarization. As shown above, we could not find a pattern for characterizing macrophages by PCR.

M1 macrophages are major participants in tumor destruction [63]. Even when we could not characterize the M1/M2 ratio for the macrophages within the tumor, we can correlate the presence of macrophages within colorectal tumors with the minor tumor development from WT mice as the presence of macrophages is related to improved survival over 60 months [59].

Macrophages are directly involved in cytokine secretion and antigen presentation. Depending on their polarization, macrophages can promote different responses in T helper cell subsets. The percentage of T cells in the tumor site was not affected, and we observed the increase of the *il-17* relative expression in MIF^−/−^ CRC, indicating that the de novo production of IL-17 by Th17 cells in the tumor was greater than that in WT tumors. Still, there were no differences in other Th17-related cytokines between MIF^−/−^ and WT CRC samples. The T helper profile has been demonstrated to be predictive of survival, treatment effectiveness, and prognosis [64]. The *il-17* gene expression in patient tumors is related to poor survival time, which correlates with the aggressiveness and the *il-17* expression of the MIF^−/−^ tumors. MIF has been well described as a promoter of the Th1 response [65]. In knockout mice, the lack of this “beneficial”-inflammation starter may favor the inflammatory Th17 pathway, but there is needed of further analysis to test this hypothesis.

In the present work, we measured immunological aspects related to the known properties of MIF on day 68, when the tumors had already developed. The tumor-promoting properties of MIF have been widely reviewed elsewhere [66], but the results previously presented demonstrate that at some point in colorectal carcinogenesis, MIF is beneficial for the host. MIF may control the carcinogenic progress by different mechanisms: by attracting macrophages to the tumor stroma, which may aid tumor destruction by phagocytosis of the transformed cells, and by regulating inflammatory response that is necessary to activate the cytotoxic activity of the CD8+ T lymphocytes.

In conclusion, MIF has a dual role in colon carcinogenesis: at the onset of tumor development, it may help to activate the immune system against tumor cells; on the other hand, increased abnormal production of MIF by the tumor cells can be used as a proliferative advantage at later tumor stages.

## Figures and Tables

**Table 1:** Sequences of the primers used to determine the immune profile of the tumors by endpoint RT-PCR. The TM and amplicon size are also shown.

Primer	Sequence	TM (°C)	Amplicon (bp)
*mif* F	5′-TGCCCAGAACCGCAACTACAGTAA-3′	60	218
*mif* R	5′-TCGCTACCGGTGGATAAACACAGA-3′		
*il-17* F	5′-TCCCTCCGCATTGACACA-3′	60	83
*il-17* R	5′-ACCGCAATGAAGACCCTGAT-3′		
*il-18* F	5′-ACTGTACAACCGCAGTAATACG-3′	58	434
*Il-18* R	5′-AGTGAACATTACAGATTTATCCC-3′		
*tnf-α* F	5′-GGCAGGTCTACTTTGGAGTCATTGC	70	195
*tnf-α* R	ACATTCGAGGCTCCAGTGAATTCG-3′		
*il-10* F	5′-ACCTGGTAGAAGTGATGCCCCAGGCA-3′	56	237
*il-10* R	5′-CTATGCAGTTGATGAAGATGTCAAA-3′		
*il-4* F	5′-CGAAGAACACAGAGAGTGAGCT-3′	58	180
*il-4* R	5′-GACTCATTCATGGTGCAGCTTATCG-3′		
*il-1β* F	5′-GAGTGTGGATCCCAAGCAAT-3′	59	520
*il-1β* R	5′-CTCAGTGCAGGCTATGACCA-3′		
*foxp3* F	5′-GGCCCTTCTCCAGGACAGA-3′	60	112
*foxp3* R	5′-GCTGATCATGGCTGGGTTGT-3′		
*arg-1* F	5′-CAGAAGAATGGAAGAGTCAG-3′	55	250
*arg-1* R	5′-CAGATATGCAGGGAGTCACC-3′		
*inos* F	5′-CTGGAGGAGCTCCTGCCTCATG-3′	62	449
*inos* R	5′-GCAGCATCCCCTCTGATGGTG-3′		
*β-Actin* F	5′-TTTGATGTCACGCACGATTTCC-3′	60	514
*β-Actin* R	5′-TGTGATGGTGGGAATGGGTCAG-3′		

## Data Availability

All data used to support the findings of this study are included within the article.

## Supplementary Materials

Supplementary Materials Supplementary Figure 1: Expression of Th17-related cytokines from colonic proteins. Healthy (blue) and CRC (red) proteins from WT and MIF^−/−^ mice were analyzed.
