# Corrigendum to “Macrophage Migration Inhibitory Factor Promotes the Interaction between the Tumor, Macrophages, and T Cells to Regulate the Progression of Chemically Induced Colitis-Associated Colorectal Cancer”

**DOI:** 10.1155/2020/2195341

**Published:** 2020-06-17

**Authors:** Thalia Pacheco-Fernández, Imelda Juárez-Avelar, Oscar Illescas, Luis I. Terrazas, Rogelio Hernández-Pando, Carlos Pérez-Plasencia, Emma B. Gutiérrez-Cirlos, Federico Ávila-Moreno, Yolanda I. Chirino, José Luis Reyes, Vilma Maldonado, Miriam Rodriguez-Sosa

**Affiliations:** ^1^Biomedicine Unit, Facultad de Estudios Superiores Iztacala, Universidad Nacional Autónoma de México (UNAM), Tlalnepantla, C.P, 54090, Mexico; ^2^Experimental Pathology Section, National Institute of Medical Sciences and Nutrition “Salvador Zubirán”, Tlalpan, C.P., 14000 Mexico City, Mexico; ^3^Epigenetics, National Institute of Genomic Medicine, Tlalpan, C.P, 14610 Mexico City, Mexico

In the article titled “Macrophage Migration Inhibitory Factor Promotes the Interaction between the Tumor, Macrophages, and T Cells to Regulate the Progression of Chemically Induced Colitis-Associated Colorectal Cancer” [1], the dot plot in Figure 6(c) for the healthy MIF^−/−^ mice inadvertently duplicated the dot plot for the MIF^−/−^ CRC mice. This was identified by the authors and is corrected by the revised figure shown in Figure 6.

## Figures and Tables

**Figure 1:**
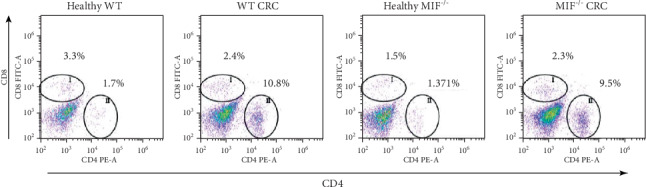
The T cell percentage is not affected by the absence of MIF. (a) CD8+ vs. (b) CD4+ T cell percentage and (c) representative dot plots of T cell staining in lamina propria from the colon of a mouse with colorectal cancer. Data are representative of three independent experiments and are plotted as the means (+SEM), *n* = 3 mice per group; ^∗^*p* < 0.05, ^∗∗^*p* < 0.01, and ^∗∗∗^*p* < 0.001.
