# Supplementary figures and images for: Macrophage Migration Inhibitory Factor Promotes the Interaction between the Tumor, Macrophages, and T Cells to Regulate the Progression of Chemically Induced Colitis-Associated Colorectal Cancer
Source: Mediators Inflamm. 2019 Jul 10;2019:2056085. doi: 10.1155/2019/2056085 (PMC6652048; doi:10.1155/2019/2056085)

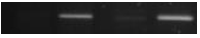

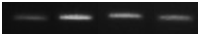

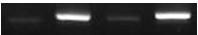


**d**

**c**

MIF-/- CRC

Healthy MIF-/-

WT CRC

Healthy WT

*il-10*

*tnf-α*

*il17*

**b**

**a**

**Supplementary figure 1**

Supplement: Supplementary Materials — Supplementary Figure 1: Expression of Th17-related cytokines from colonic proteins. Healthy (blue) and CRC (red) proteins from WT and MIF−/− mice were analyzed. [file 2056085.f1.docx]
